# Physiological and Metabolic Responses of Wheat (*Triticum aestivum* L.) after One-Generation Exposure to Perfluorooctanesulfonic Acid (PFOS)

**DOI:** 10.1021/acsagscitech.4c00722

**Published:** 2025-03-07

**Authors:** Olamide R. Ogundele, Mary Fakunle, Riley Pope-Buss, Jacob Churchman, Blessing Akinwande, Naum Kirwa, Polycarp C. Ofoegbu, Cyren M. Rico

**Affiliations:** Missouri State University, 901 S National Ave, Springfield, Missouri 65897, United States

**Keywords:** emerging contaminants, epigenetics, generational exposure, metabolomics, PFAS, response memory

## Abstract

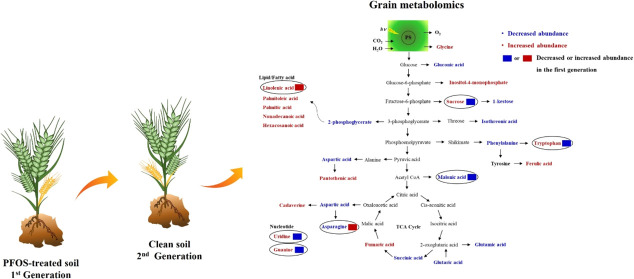

The pattern of plant responses, particularly on the seeds/grains metabolite profile, after generational exposure to contaminants is not well documented. Seeds from wheat cultivated in soil amended with PFOS at 0 and 25 mg/kg in the first generation were grown in clean soil to produce daughter plants and seeds in the second generation and assigned treatment combinations of 0–0 mg/kg PFOS and 25–0 mg/kg PFOS. Plant stress and responses including growth and biomass production, chlorophyll content, lipid peroxidation, and enzyme activity were measured over a short exposure period (21 days growth period). Biomass yields, elemental concentration, and grain metabolites were also measured after a long exposure period (92 days growth period). The daughter plants exhibited decreased chlorophyll content and lipid peroxidation in a short exposure period. The elemental concentrations were mostly not affected except for changes in microelements, except B, in the grains. In the metabolomics analysis, grains harvested from plants previously exposed to PFOS (i.e., 25–0 mg/kg PFOS) showed increased abundances of sucrose, linolenic acid, tryptophan, inositol-4-monophosphate, and ferulic acid, perhaps in response to adaptation to former stress. The current findings seem to suggest that one-generation exposure to PFOS does not cause detrimental effects on the next generation after the cessation of exposure. The results provide insights into the effects of generational exposure of plants to PFOS.

## Introduction

Perfluorinated and polyfluorinated alkyl substances (PFAS) make up a class of anthropogenic chemicals, some of which have been in use for over 50 years.^1,2^ The environmental persistence of these compounds containing carbon–fluorine (C–F) bonds is attributed to their high-energy bond, which confers resistance to hydrolysis, photolysis, microbial degradation, and animal metabolism.^3^ Perfluorooctanesulfonic acid (PFOS) is a historically and widely used PFAS that exhibits hydrophobic and oleophobic qualities and hence is utilized in a variety of commercial and consumer products, such as surface protection materials, floor polishes, shampoos, fire-resistant coatings, stain repellents, insecticides, lubricants, paints, and pharmaceuticals.^4−6^ PFOS is pervasive, biologically persistent, hazardous to wildlife and humans, and stable and resistant to degradation—characteristics that have led to its worldwide emergence as a novel environmental pollutant.^4,7^ The discovery of high concentrations of PFOS in agricultural soils has raised concerns about the possibility of PFOS transfer to crops and subsequent phytotoxicity, which may pose a threat to animal and human health.^6,8,9^ PFOS production has been largely phased out; however, concerns remain about the potential long-term exposure to PFOS due to its environmental persistence.^10^

Empirical evidence shows that exposure of animals to PFAS may have heritable impacts on future generations via epigenetic pathways.^11^ However, little or no research has been done on the generational effects of PFAS in plants. Epigenetics refers to enduring changes in a cell’s transcriptional capabilities, which need not necessarily be heritable, as well as heritable modifications in gene activity and expression across subsequent cell generations or individuals.^12−16^ Without causing changes to an organism’s DNA sequences, these modifications show mitotic stability and may even express meiotically inheritable features for several upcoming generations. In the context of plant biology, the phenotype or environmental conditions of the parent plants, in addition to environmental factors, genotype, and their complex interplay, have a substantial influence on phenotypic alterations.^12,13^

Generational exposures of plants to environmental contaminants have not yet been widely studied yet. Ogunkunle et al.^17^ summarized the elemental changes in progeny of seeds generationally exposed to engineered nanonparticles. These researchers noted that engineered nanoparticles could affect elemental accumulation in the grains of progeny plants after generational exposure to the contaminant, although the mechanism is not known. In our studies on the exposure of three generations of wheat to cerium oxide nanoparticles, we found a successive decrease in nicotianamine in progeny grains that coincided with decreased concentration of Fe in grains perhaps due to the function of nicotianamine in producing mucigineic acid for elemental accumulation in plants.^18^ Shimalina et al.^19^ also investigated the generational effects of ionizing radiation on the performance of *Plantago major* plants and found varying degrees of influence on progeny plants depending on the extent of exposure. Evidence indicates persistent changes in gene regulation in rice (*Oryza sativa*) even after the removal of heavy metal stress.^16^ Recently, Zhang et al.^20^ reported that prior exposure to PFAS affects the ability of soybean (*Glycine max*) to take up and accumulate PFAS in the next generation, i.e., some PFAS showed increased uptake in second-generation plants. On the other hand, Liu et al.^21^ reported that the uptake of Cu or As in second-generation rice plants was not dependent on the parental exposure to Cu nanoparticles and As. However, omics-based investigations on the generational effects of contaminants on grain quality are not widely available in the literature.

The literature is replete with studies demonstrating the metabolomic changes in plants exposed to abiotic stresses, but very few studies were directed toward understanding changes in the metabolite profile of grains. Moreover, the carryover and generational effects of stress on grain metabolites are rarely reported in the literature and are therefore poorly understood. Metabolites are photosynthates produced from photosynthetic processes during plant growth and development. These photosynthates are distributed to different parts of the plant depending on the growth stage, and in the case of cereals, the photosynthates are delivered and stored in seeds and grains during the reproductive development at the latter stage of plant life. The synthesis of metabolites in the leaves and, ultimately, the transport and storage of the metabolites in the seeds/grains are a result of the plant’s response to environmental conditions or stress.^22^ Thus, the extent of metabolomic dysregulation provides insight into how the stress experienced by the parents influences the biochemical makeup and physiology of daughter plants.

Wheat (*Triticum aestivum* L.) is a dietary staple for a significant population worldwide and serves as a crucial source of carbohydrates, dietary fiber, phytochemicals, and vitamin B.^48^ Several studies have investigated its uptake of a combination of PFAS under short exposure scenarios, mostly in hydroponics (and a few in soil).^6^ While previous research has investigated how wheat responds to PFOS in soil over both short-term and complete lifecycles at the physiological, biochemical, and molecular levels, this study will focus on the effects of PFOS across two generations in wheat. There are very few studies on how prior exposure to stress affects the growth pattern or the grain metabolite profile of plants with or without the presence of the stress in the succeeding generation. It is not clear whether prior stress exposure affects or not the delivery and subsequent storage of photosynthates in the progeny seeds. The persistence of the phenotypic effects across generations also remains insufficiently understood. Here, we focused our efforts on documenting the influence of PFOS on plant photosynthetic and stress response, biomass yields, and grain metabolome in second-generation plants removed from PFOS stress. The hypothesis tested was that the stress response, productivity, and grain metabolite levels will be lower in progeny of PFOS-stressed plants compared to stress-free plants.

## Materials and Methods

### Experimental Design

Wheat exposed to 0 or 25 mg of PFOS per kilogram of soil in the first generation provided seeds that were cultivated in clean soil (0 mg/kg) to produce second-generation plants. The treatments were 0–0 mg/kg PFOS or 25–0 mg/kg PFOS, which indicate 0 or 25 mg/kg PFOS in the first generation and 0 mg/kg PFOS in the second generation. The experiment was repeated twice to investigate plant responses at both short-term or juvenile stage (21 days) and full-lifecycle or long-term (70 days) exposures. The soil used in this study was Pro-mix high-porosity potting soil (PRO-MIX HP), which is good for preventing water stagnation and promoting soil aeration. Two sets of pots were arranged: one set comprising 12 pots, each containing 700 g of soil, for long-term exposure; and another set comprising 12 pots, each holding 300 g of soil, for short-term exposure. The pots were systematically organized into replicates, with 6 pots (replicates) allocated per treatment. The pots were arranged in a 3 × 4 pot layout and their position rotated every 3 days. Millipore water was added to all pots as needed to keep the soil moist, while fertigation was done by adding Yoshida Nutrient Solution regularly.^23^

### Biochemical Assays

The determination of chlorophyll content involved immersing fragments (100 mg) from the youngest leaves in 5 mL of 70% ethanol for 24 h in the dark. The absorbance of the supernatant was read using a Cary 60 Ultraviolet–visible spectrometer, and the contents of chlorophyll a and b were quantified following the procedure employed by Rico et al.^24^ In the analysis of lipid peroxidation, wheat leaves (0.5 g) underwent cryogenic grinding in liquid nitrogen and subsequent homogenization in trichloroacetic acid (TCA). The resulting homogenates were subjected to centrifugation, and a solution comprising the supernatant, thiobarbituric acid (TBA), and butylated hydroxytoluene (BHT) was subjected to heating at 95 °C for 30 min. The heated mixture was cooled in ice and centrifuged for 15 min at 5000 rpm. The absorbance of the supernatant was measured, and the quantification of lipid peroxidation was determined by measuring thiobarbituric acid reactive substances (TBARS) using the formula a μM TBARS/g = [(Abs_532_ – Abs_600_)/ε]/mass of the sample, where ε represents the molar absorptivity coefficient (155 mol^–1^ cm^–1^ L), and Abs_532_ and Abs_600_ correspond to the absorbance readings at 532 and 600 nm, respectively.

### Elemental Uptake Analysis

In this study, both the ground plant samples (roots, shoots, grains) and peach leaves as the reference standard (NIST 1547, Gaithersburg, MD) underwent digestion in 5 mL of plasma-grade nitric acid (SCP Sciences, Champlain, NY) by employing a microwave digester (CEM Mars 6TM, Matthews, NC). The digestates were diluted to 50 mL solution before preparing 5 times dilution for ICP-MS (ICP-MS, Agilent Technologies 7900) analysis. Percent recoveries of elements from the peach reference standard were 90–110%.

### Metabolomics Analysis

Metabolomics was performed at the West Coast Metabolomics Center, University of California Davis, following the method by Fiehn et al.^25^ The analysis report gave *m*/*z* values for peak heights instead of peak areas to improve the precision for less-abundant metabolites. Only metabolites with 95% confidence level were used for further analysis, i.e., partial least-squares-discriminant analysis (PLS-DA), in MetaboAnalyst 5.0 (https://www.metaboanalyst.ca). All metabolites were assigned a variable importance in projection value (VIP) by PLS-DA with a VIP value >1 implying that a metabolite contributed to group-clustering differences.^26^ Lastly, pathway analysis was performed using the metabolites with VIP > 1.

### Statistical Analysis

All data were analyzed using a statistical analysis software package (SAS Institute, Cary, NC). One-way analysis of variance (ANOVA) followed by least significant difference (LSD) analysis was performed to determine the statistical differences between treatments.

## Results and Discussion

### Biochemical Changes at Juvenile Stage

**Figure 1:**
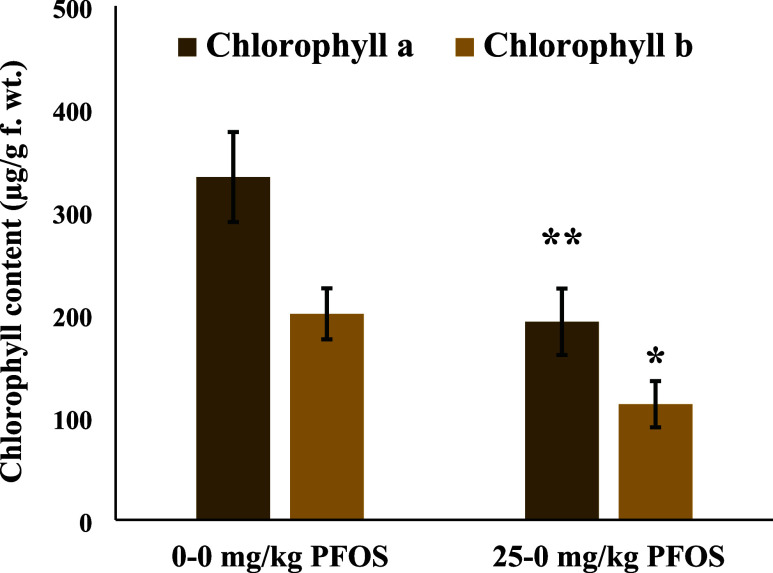
Chlorophyll concentration in wheat previously exposed to perfluorooctanesulfonic acid (PFOS) at juvenile stage (21 days). Values are mean ± SE (*n* = 6). * and ** indicate statistical difference between treatments at *p* < 0.10 and *p* < 0.05, respectively.

**Figure 2:**
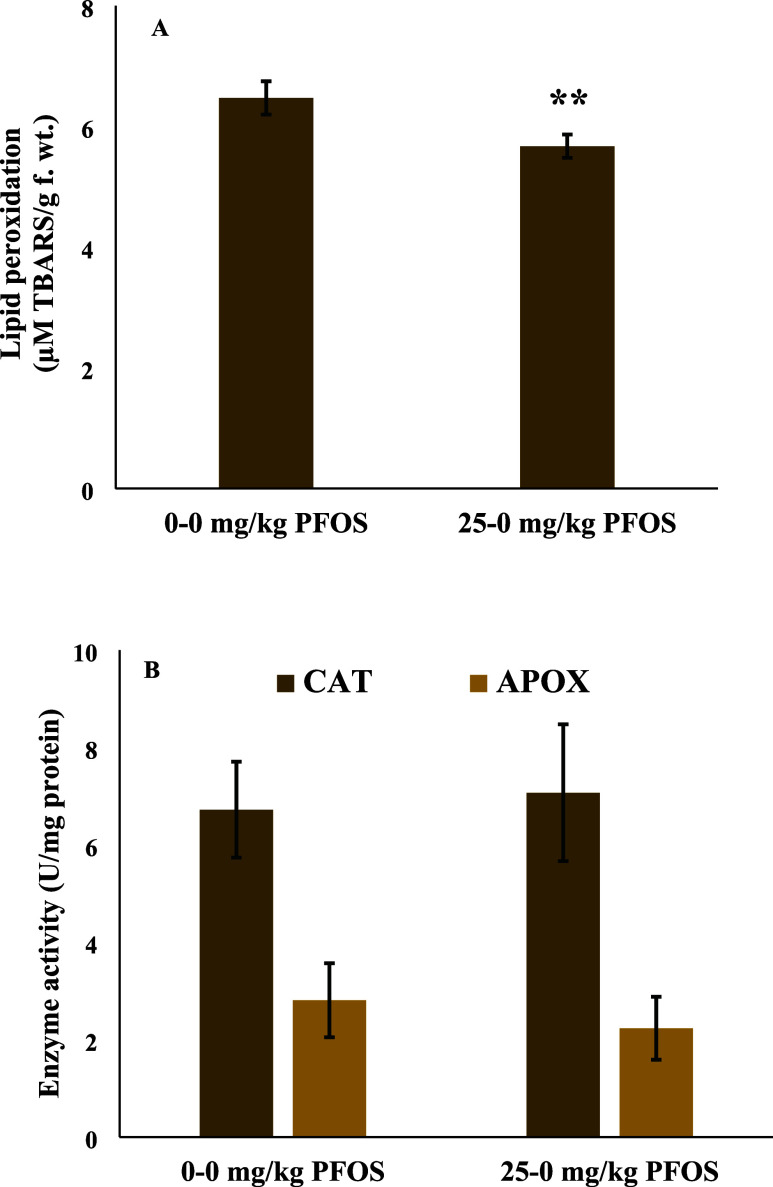
(A) Lipid peroxidation and (B) enzyme activities in wheat previously exposed to perfluorooctanesulfonic acid (PFOS) at juvenile stage (21 days). Values are mean ± SE (*n* = 6). ** indicates statistical difference between treatments at *p* < 0.10.

There were no major persistent generational biochemical changes that were noticeable in the juvenile growth stage of the plant. With the exception of pigment production, wherein chlorophyll a and b concentrations decreased in 25–0 mg/kg PFOS (193 and 113 μg/g, respectively) compared to 0–0 mg/kg PFOS (334 and 200 μg/g, respectively) (Figure 1), the other biochemical assays did not show the carryover effects of parental exposure to PFOS. Even the measure of stress via lipid peroxidation (LPOX) showed that the plants previously exposed to PFOS in the first generation (25–0 mg/kg PFOS) had lower oxidative stress (5.67 μM TBARS/g) than the control (0–0 mg/kg PFOS) plants (6.47 μM TBARS/g) (Figure 2). The nonsignificant decreasing trend in the catalase (CAT) and ascorbate peroxidase (APOX) activities supports the LPOX data and therefore lower oxidative stress in 25–0 mg/kg PFOS plants compared to 0–0 mg/kg PFOS (Figure 2). The negative effects of previous-generation exposure to PFOS on pigment production at the juvenile growth stage of the second-generation plants was not in agreement with our data from the first-generation study wherein chlorophyll production or LPOX was not affected by 25 mg/kg PFOS compared to control (0 mg/kg).^5^

Medina-Velo et al.^27^ also performed a generational exposure of soybean to ZnO nanoparticles (ZnO-NPs) involving an experimental setup similar to the current study (i.e., second-generation plants were grown without exposure to stress). They found that prior exposure to ZnO-NPs (500–0 mg/kg ZnO-NPs) did not affect the activities of CAT, APOX, and superoxide dismutase (SOD) enzymes when compared to those from control plants (0–0 mg/kg ZnO-NPs). Wang et al.^28^ also found no effects of previous-generation exposure to cerium oxide nanoparticles (CeO_2_–NPs) on oxidative stress via H_2_O_2_ production in second-generation tomato seedlings grown in clean soil (10–0 mg/L CeO_2_–NPs) compared to control plants (0–0 mg/L CeO_2_–NPs). In another generational study with a similar experimental setup to the current study, Shimalina et al.^19^ found that *P. major* exhibited oxidative stress via LPOX even after removal of stress in second- and third-generation plants, but only when the parent plants had been exposed to a very high concentration of ionizing radiation. Nonetheless, they found that CAT and superoxide dismutase (SOD) enzyme activities did not sustain a consistent trend across second- and third-generation stress-free plants. Overall, the removal of stress in cultivating the progeny from stressed parent plants seems to allow plants to grow without showing signs of stress. This finding is significant considering that plant’s early-life stages are sensitive to environmental stress experienced in the previous generation.^12,15,21^

### Plant Growth and Biomass

**Figure 3:**
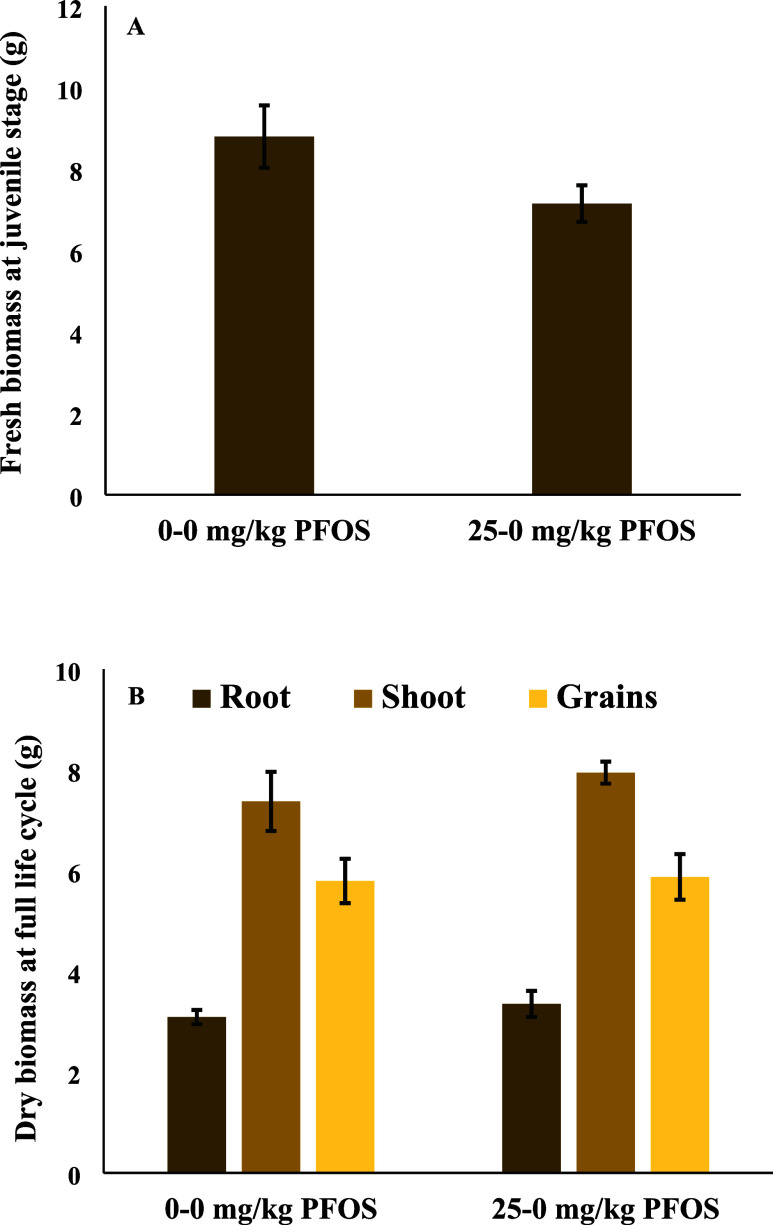
(A) Fresh biomass at juvenile stage (21 days) and (B) dry biomass of roots, shoots, and grains in the full life cycle (70 days) of wheat previously exposed to perfluorooctanesulfonic acid (PFOS). Values are mean ± SE (*n* = 6). * indicates statistical difference between treatments at *p* < 0.05.

The current findings highlight the importance of full-life cycle study, rather than short exposure study in juvenile or seedling stage, to provide a more robust understanding of plants’ responses to contaminants.^12,13,15^ The results demonstrated that parental exposure to 25 mg/kg PFOS and removal of stressor (i.e., PFOS) in the second-generation plants did not affect the agronomic and yield performance of wheat (Figure 3). Plant height did not change between treatments (0–0 mg/kg PFOS versus 25–0 mg/kg PFOS) (data not shown). Similarly, plant biomass yields were not affected although a nonsignificant decreasing trend in whole plant biomass seemed to follow the lower chlorophyll content in the short exposure study. The data showed that although plants had experienced PFOS in the previous generation (25–0 mg/kg PFOS), the whole plant biomass for a short exposure period or biomass yields (root, shoot, and grain biomass) in the full-life cycle study were still similar to those of the control plants (0–0 mg/kg PFOS) (Figure 3).

The results from our first-generation study also showed that 25 mg/kg PFOS had no significant effects on the root, shoot, and grain biomass yields of wheat compared to the control plants (0 mg/kg PFOS) in short- and long-term exposure regimes.^5^ However, the apparent absence of toxicity or lack of negative responses in growth and biomass production during the seedling stage and even at full maturity does not completely agree with the poor seed quality from first-generation treatment (25 mg/kg PFOS). First-generation seeds from 25 mg/kg PFOS had lower sugar abundance (e.g., glucose, glucose-6-phosphate, fructose-6-phosphate, sucrose, trehalose) compared to control (0 mg/kg PFOS),^5^ and the low sugar abundance should have caused delay or growth impediment. The current findings were expected since wheat plants were not exposed to a stressor (i.e., PFOS) during the second-generation study, and parental exposure to 25 mg/kg PFOS could perhaps be considered low to induce generational effects on the agronomic or yield characteristics of plants. Another related generational study by Shimalina et al.^19^ found consistent effects of root growth reduction in second- and third-generation *P. major* plants that were grown free of stress (i.e., ionizing radiation), showing stable transgenerational effects on root growth.

Generational exposure of wheat to CeO_2_–NPs showed the effects of parental exposure to the nanoparticles on the performance of the succeeding generation wherein plant height and number of open or dry spikes in plants increased more noticeably in 500 mg/kg CeO_2_–NPs than in 250 mg/kg CeO_2_–NPs.^29^ Despite these positive changes, parental exposure to CeO_2_–NPs did not affect the grain yield of daughter plants at harvest.^29^ Medina-Velo et al.^27^ also found no effect of parental exposure to ZnO-NPs (500–0 mg/kg ZnO-NPs) on the yield of daughter plants cultivated in soil free of ZnO-NPs (0–0 mg/kg ZnO-NPs). In contrast, studies found effects of parental exposure to CeO_2_–NPs (10–0 mg/L CeO_2_–NPs) via slower growth and smaller biomass production in tomato *(Lycopersicon)* grown in clean soil (0–0 mg/L CeO_2_–NPs).^28^ Another study also found that parental exposure to CuO-NPs and As had effects on the seed germination and seedling growth of offspring generation.^21^ Overall, the data suggest that PFOS does not constitute a threat to growth and productivity when wheat is exposed to a low concentration of 25 mg/kg PFOS for one generation and grown in PFOS-free soil in the second generation.

### Elemental Contents in Wheat

Parental exposure to PFOS did not cause large modifications of the concentration of macro- and microelements in the different parts of wheat plants. The roots and shoots harvested from wheat previously exposed to PFOS (25–0 mg/kg PFOS) did not exhibit changes in elemental concentration compared to those from control plants not exposed to PFOS in both generations (0–0 mg/kg PFOS) (Table 1). However, B concentration decreased by roughly 47% in wheat grains harvested from 25 to 0 mg/kg PFOS compared to 0–0 mg/kg PFOS (Table 1). The first-generation study also showed that macro- and microelement concentrations in 25 mg/kg PFOS treated grains did not change significantly compared to 0 mg/kg PFOS with the exception of grain Fe concentration, which increased tremendously in 25 mg/kg PFOS compared to control (0 mg/kg).^5^ It is not clear how parental exposure to PFOS would cause modifications in B concentration in wheat grains. But just like in the first-generation study, this result indicates minimal effects of 25 mg/kg PFOS on the elemental concentrations of second generation grown in clean soil.

**Table 1:** Elemental Concentrations in Wheat Generationally Exposed to Perfluorooctanesulfonic Acid (PFOS)^a^

	0–0 mg/kg PFOS	25–0 mg/kg PFOS
Grains		
Mg	1597 ± 192a	1591 ± 117a
P	5465 ± 794a	5507 ± 388a
S	5361 ± 697a	5087 ± 381a
K	645 ± 83a	638 ± 31a
Ca	448 ± 62a	469 ± 28a
Mn	15 ± 2a	13 ± 1a
Fe	76 ± 18a	75 ± 7a
B	8.06 ± 1.66a	4.31 ± 0.22b
Shoots		
Mg	3615 ± 133a	3988 ± 341a
P	4871 ± 508a	4232 ± 607a
S	4991 ± 272a	6004 ± 156a
K	21701 ± 1576a	22276 ± 1802a
Ca	8032 ± 491a	9180 ± 849a
Mn	24 ± 3a	24 ± 2a
Fe	178 ± 55a	161 ± 39a
B	18.9 ± 1.5a	20.3 ± 2.1a
Roots		
Mg	927 ± 88a	905 ± 103a
P	1283 ± 144a	1453 ± 263a
S	4767 ± 136a	4821 ± 133a
K	2665 ± 397a	2550 ± 203a
Ca	7836 ± 770a	7649 ± 968a
Mn	32 ± 5a	56 ± 30a
Fe	542 ± 134a	618 ± 208a
B	10.9 ± 3.1a	5.9 ± 0.2a

a Values are means ± SE (*n* = 6). Different letters indicate statistical difference between treatments.

In general, the shoot to grain elemental movement and finally elemental storage in grain are complex physiological processes. The generational treatment in this study even complicates the understanding of modifications in the elemental concentrations in grains. There are several studies on the impacts of generational exposures to contaminants on the elemental concentration in plants, but very few studies have focused on elemental modifications in seeds or grains. For example, our study on CeO_2_–NPs in wheat showed that 250–0 or 500–0 mg/kg CeO_2_–NPs reduced Al, Fe, and Mn concentrations in grains compared to control (0–0 mg/kg CeO_2_–NPs).^29^ On the other hand, Medina-Velo et al.^27^ found no significant changes in elemental concentrations in the seeds of kidney beans in 500–0 mg/kg ZnO-NPs vs 0–0 mg/kg ZnO-NPs.

### Metabolomic Changes in Grains

**Figure 4:**
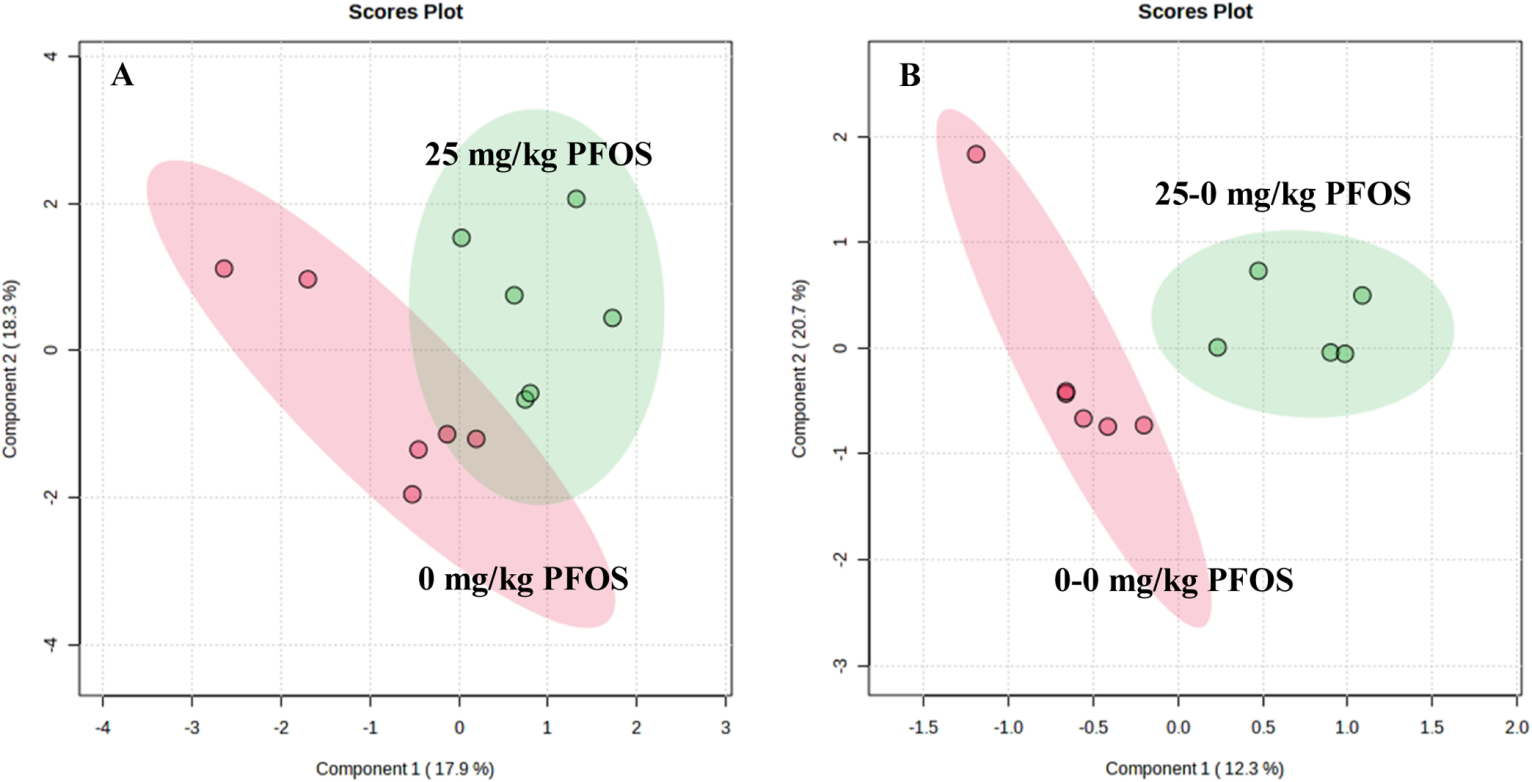
Partial least-squares discriminant analysis (PLS-DA) of grains of (A) wheat exposed to perfluorooctanesulfonic acid (PFOS) in the first generation and (B) wheat previously exposed to PFOS but grown in clean soil in the second-generation study.

The grains’ metabolomics data in the first-generation study (25 mg/kg PFOS vs 0 mg/kg PFOS) and the second-generation study (25–0 mg/kg PFOS vs 0–0 mg/kg PFOS) were analyzed separately using MetaboAnalyst (https://www.metaboanalyst.ca/) since the metabolites detected from the two studies did not match exactly. The score plots revealed a much larger separation between treatments in the second-generation study (i.e., no overlap in ellipses) than in the first-generation study, indicating a greater modification in metabolite composition in 25–0 mg/kg PFOS vs 0–0 mg/kg PFOS than in 25 mg/kg PFOS vs 0 mg/kg PFOS (Figure 4). The results from both studies indicate that PFOS altered the grain metabolite composition, which, interestingly, was more amplified in the second generation, as demonstrated visually by the large separation or nonoverlapping between treatments (i.e., ellipses) (Figure 4).

**Figure 5:**
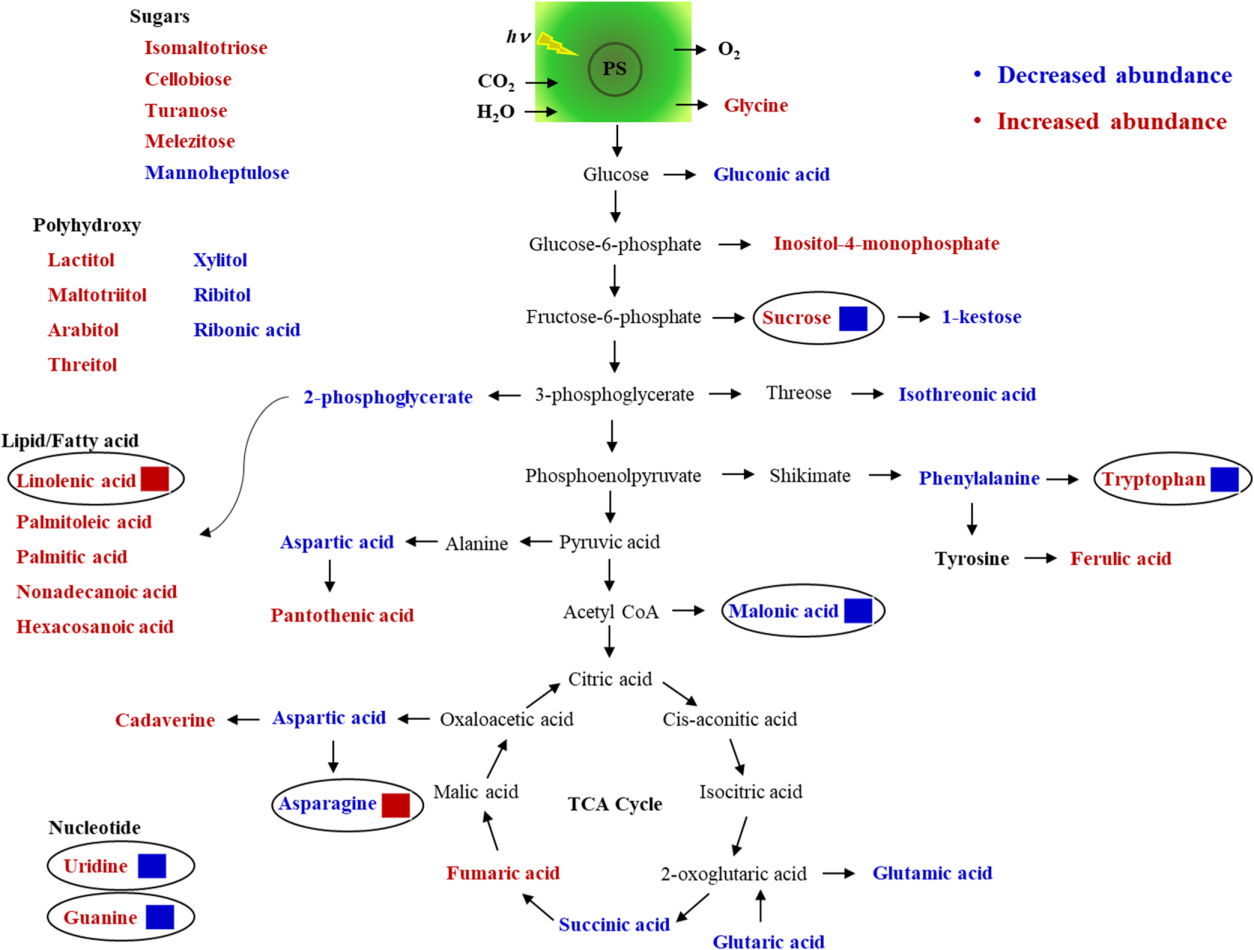
Metabolic pathway showing the changes in metabolite abundance (VIP score >1) in wheat grains harvested from plants exposed to perfluorooctanesulfonic acid for the full life cycle. Metabolites in red or blue font signify increase or decrease, respectively, in 25–0 mg/kg PFOS compared to 0–0 mg/kg PFOS. The red or blue boxes indicate increase or decrease, respectively, in 25 mg/kg PFOS compared to 0 mg/kg PFOS during the first-generation study.

The metabolomic data suggest that parental exposure to 25 mg/kg PFOS could alter the metabolite composition of grains even when the daughter plants did not encounter PFOS stress. There were 188 metabolites identified in the second-generation study using the KEGG Pathway Database (Table 2 and Figure 5). However, only 39 metabolites had a VIP > 1, 23 of which increased, while the other 15 metabolites decreased, in abundance in 25–0 mg/kg PFOS versus 0–0 mg/kg PFOS (Figure 5). These changes in trends explain why the treatments (i.e., ellipses) of 0–0 mg/kg PFOS and 25–0 mg/kg PFOS did not overlap in the second-generation study, whereas the ellipses of 25 mg/kg PFOS and 0 mg/kg PFOS overlapped in the first-generation study (Figure 4). Interestingly, the top biochemical pathways affected mostly involved amino acid metabolites (asparate, asparagine, glutamate, glycine, and phenylalanine), TCA cycle metabolites (fumarate and succinate), and a biogenic polyamine (cadaverine; Table 3).

**Table 2:** Metabolites in Wheat Grains that Showed VIP value >1.0

	metabolites	0–0 mg/kg PFOS	25–0 mg/kg PFOS	percent change (%)
sugars	isomaltotriose	19482 ± 3909	27303 ± 4081	+40
	cellobiose	149971 ± 32179	211125 ± 25853	+41
	sucrose	1737535 ± 494791	2272623 ± 420930	+31
	turanose	4950 ± 212	6427 ± 302	+30
	melezitose	30207 ± 5197	39634 ± 5598	+31
	mannoheptulose	6455 ± 1286	5399 ± 1579	–16
	1-kestose	1640891 ± 354721	996056 ± 196969	–39
polyhydroxy	lactitol	61766 ± 15787	120868 ± 11337	+95
	maltotriitol	48473 ± 16730	63076 ± 3420	+30
	arabitol	114419 ± 18873	179805 ± 20015	+57
	threitol	16855 ± 1963	184752 ± 169778	+996
	ribitol	21800 ± 2968	20796 ± 6458	–5
	xylitol	71335 ± 10843	48393 ± 10115	–32
	isothreonic acid	5953 ± 594	4376 ± 440	–26
	ribonic acid	14775 ± 1030	10984 ± 2203	–25
amino acids	glycine	145891 ± 24304	168187 ± 4492	+15
	tryptophan	216518 ± 91270	320920 ± 124027	+48
	aspartic acid	884001 ± 141393	634812 ± 92624	–28
	asparagine	301934 ± 47027	228096 ± 56937	–24
	glutamic acid	182930 ± 25115	145971 ± 26956	–20
	phenylalanine	52239 ± 5243	41442 ± 6933	–20
fatty acids	linolenic acid	30834 ± 5765	40854 ± 2291	+33
	palmitoleic acid	4967 ± 722	6400 ± 373	+29
	palmitic acid	876761 ± 89332	1144879 ± 100803	+31
	nonadecanoic acid	12422 ± 1348	16376 ± 864	+32
	cerotinic acid (hexacosanoic)	13050 ± 2673	15148 ± 1322	+16
organic acids	ferulic acid	2138 ± 246	2963 ± 550	+39
	fumaric acid	102393 ± 7843	142279 ± 17774	+39
	inositol-4-monophosphate	9004 ± 1319	12488 ± 1117	+39
	cadaverine	12781 ± 2660	16664 ± 3217	+30
	pantothenic acid (vit b-5)	7706 ± 1534	10813 ± 739	+40
	malonic acid	2829 ± 336	1803 ± 565	–36
	gluconic acid	13051 ± 2486	9843 ± 2595	–25
	glutaric acid	3363 ± 274	2154 ± 299	–36
	β-glycerolphosphate	9094 ± 1701	5185 ± 1350	–43
	succinic acid	88312 ± 12981	66551 ± 8793	–25
nucleobase/sides	uridine	431045 ± 25265	569838 ± 28631	+32
	guanine	23919 ± 5034	31272 ± 4622	+31

Alterations in the abundances of these metabolites in the grains caused differences or separations between PFOS treatments. Values are means ± SE (*n* = 6).

**Table 3:** Perturbed Biological Pathways in Wheat Generationally Exposed to Perfluorooctanesulfonic Acid (PFOS)

pathways	*p*	match status	involved metabolites
alanine, aspartate, and glutamate metabolism	9.8 × 10^–6^	5/22	aspartate, asparagine, glutamate, fumarate, succinate
arginine biosynthesis	2.0 × 10^–3^	3/18	glutamate, aspartate, fumarate
cyanoamino acid metabolism	5.3 × 10^–3^	3/25	asparagine, glycine, aspartate
glutathione metabolism	6.6 × 10^–3^	3/27	glycine, glutamate, cadaverine
tropane, piperidine, and pyridine alkaloid biosynthesis	7.1 × 10^–3^	2/9	cadaverine, phenylalanine
glyoxylate and dicarboxylate metabolism	8.1 × 10^–3^	3/29	glycine, glutamate, succinate

### Grain Metabolites Affected Generationally

Despite the metabolomic changes induced by prior exposure to PFOS, the data suggest that the removal of PFOS after one generation exposure did not seem to show consistent adverse carryover effects on grain metabolites. This is supported by the data that show only seven metabolites with VIP > 1 were detected in both first- and second-generation studies (sucrose, asparagine, tryptophan, linolenic acid, malonic acid, uridine, and guanine), and only linolenic acid and malonic acid exhibited consistent trends in response across generations (Figure 5).

Sucrose decreased in abundance by 16% when plants were exposed to PFOS in the first generation (25 mg/kg PFOS vs 0 mg/kg PFOS)^5^ but increased in abundance by 31% in 25–0 mg/kg PFOS compared to 0–0 mg/kg PFOS (Table 2). Sucrose decreased in the first-generation study due to decreases in glucose and fructose synthesis in the PFOS-treated grains, but since these metabolites were not affected in the second-generation study, the 25–0 mg/kg PFOS plants synthesized and stored more sucrose than the control (0–0 mg/kg PFOS). Tryptophan decreased in abundance by 26% in 25 mg/kg of PFOS compared to 0 mg/kg of PFOS, but it increased in abundance by 48% in 25–0 mg/kg of PFOS compared to control (Figure 5). The data showed that tryptophan synthesis was favored in 25–0 mg/kg PFOS vs 0–0 mg/kg PFOS perhaps due to greater conversion of the precursor phenylalanine to tryptophan since phenylalanine’s abundance decreased in 25–0 mg/kg PFOS compared to 0–0 mg/kg PFOS (Figure 5). Asparagine increased in abundance by 123% in 25 mg/kg of PFOS versus 0 mg/kg of PFOS but decreased in abundance by 24% in 25–0 mg/kg of PFOS versus 0–0 mg/kg of PFOS (Table 2 and Figure 5). In 25–0 mg/kg PFOS grains, the abundance of asparagine decreased compared to the control (0–0 mg/kg PFOS) presumably due to the decreased abundance of aspartic acid (Figure 5). Linolenic acid increased in 25 mg/kg PFOS and 25–0 mg/kg PFOS by 216 and 32%, respectively, compared to their respective controls. On the other hand, malonic acid is the only metabolite that had consistent decrease in abundance in 25 mg/kg PFOS vs 0 mg/kg PFOS and 25–0 mg/kg PFOS vs 0–0 mg/kg PFOS by roughly the same amount (40 and 36%, respectively). Uridine and guanine decreased in abundance by 11 and 16%, respectively, in the first-generation study (25 mg/kg PFOS vs 0 mg/kg PFOS) but increased in abundance by 32 and 31%, respectively, in the second-generation study (25–0 mg/kg PFOS vs 0–0 mg/kg PFOS). These DNA/RNA metabolites showed directly related increases or decreases in abundances with sugars (especially sucrose) in both first and second-generation studies.

### Changes in Other Grain Metabolites

#### Sugar Metabolites

Metabolomics analysis revealed that the abundances of sugar and polyhydroxy (sugar alcohol and sugar acid) metabolites were significantly altered in the second-generation study (Figure 5). This is an intriguing observation since the PFOS stress was introduced during the parent generation only, yet it exhibited influence over the translocations of sugars from leaves (source) to grains (sink). The major sugar metabolites (sucrose, cellobiose, turanose, melezitoze, and isomaltotriose) increased in abundances by 31, 41, 30, 31, and 40%, respectively, in 25–0 mg/kg PFOS versus 0–0 mg/kg PFOS. The affected sugars are disaccharides (sucrose, cellobiose, and turanose) or trisaccharides (melezitoze and isomaltotriose) of glucose and/or fructose even though glucose or fructose abundance was not affected by the treatment. Turanose, which is an isomer of sucrose, is the product of acid hydrolysis of melezitose.^30^ Increased production of sucrose and turanose has been observed in cotton (*Gossypium hirsutum*) under salt stress^31^ and in grains of a rice (*O. sativa*) variety that is tolerant to 2,2′,4,4′-tetrabromodiphenyl ether (PBDE).^32^ Notably, 1-kestose (a fructooligosaccharide composed of one unit of sucrose and two units of fructose and the most common kestose in plants) decreased in 25–0 mg/kg PFOS compared to 0–0 mg/kg PFOS. This is perhaps because sucrose became the preferred storage of sugar and the sucrose to 1-kestose conversion was inhibited (Figure 5). This observation was also supported by simultaneous increases in the abundances of sucrose and cellobiose, which signifies upregulation in starch and sucrose metabolism. Mannoheptulose, a seven-carbon sugar (C7) metabolite, was the only monosaccharide that was affected, and its abundance decreased by 16% in 25–0 mg/kg PFOS compared to 0–0 mg/kg PFOS. Mannoheptulose serves as a carbon sink that is highly mobilized in plants to meet the nutrient requirements for growth and development.^33^ It is also believed that C7 sugar competes with sucrose synthesis,^34^ and in light of increased sucrose abundance, this could perhaps explain the decreased abundance of mannoheptulose.

#### Lipid Metabolites

Similar to the sugar metabolites, important lipid metabolites (VIP > 1) also significantly increased in abundance in the second-generation study (Figure 5). Increases in fatty acid metabolites, especially the unsaturated ones, as a consequence of prior exposure to PFOS suggest that parental exposure could exert impact on the energy metabolism and stress regulation in daughter plants. Palmitoleic, linolenic, palmitic, nonadecanoic, and hexacosanoic (cerotinic acid) acids increased in abundance by 29, 32, 31, 32, and 16% in 25–0 mg/kg PFOS compared to 0–0 mg/kg PFOS (Table 2 and Figure 5). Palmitoleic and linolenic acids are unsaturated fatty acids, while palmitic, nonadecanoic, and hexacosanoic are saturated fatty acids. Cereal grains harvested from plants exposed to different contaminants (cerium oxide nanoparticles, PFOS, PBDE) have shown dysregulated abundance of lipid metabolites.^5,18,32^ The first-generation study also showed increased abundances of oleic and linolenic acids when exposed to 25 mg/kg PFOS.^5^ The current understanding on lipid metabolites in stressed plants is that increased abundance of these metabolites is an indicator of oxidative stress,^35^ but since the plants were not exposed to stress in the second generation, the conventional understanding that changes in fatty acid metabolites are a response to oxidative stress should be revisited. The question whether increased abundance in lipid metabolites, especially unsaturated fatty acids, means parental exposure to PFOS exerts stress on the progeny generation also requires further investigation.

#### Amino Acid Metabolites

The second-generation study showed that only proteinogenic amino acids (i.e., aspartic acid, asparagine, glutamine, phenylalanine, tryptophan, and glycine) were affected as opposed to both proteinogenic and nonproteinogenic amino acids impacted in the first-generation exposure study (Figure 5). Glutamic acid, aspartic acid, asparagine, and phenylalanine decreased in abundance in 25–0 mg/kg of PFOS compared to 0–0 mg/kg of PFOS by 20, 28, 24, and 21%, respectively. On the other hand, tryptophan and glycine increased in abundance in 25–0 mg/kg PFOS compared to control by 48 and 31%, respectively. These data are generally very similar to our first-generation PFOS study in wheat wherein generally PFOS increased the abundance of polar amino acids and decreased the abundance of nonpolar amino acids.^5^ This observation suggests the selectivity of daughter plants previously exposed to PFOS toward amino acid metabolism in wheat and thereby affecting the accumulation of amino acid in grains. The current results also showed that daughter grains had much fewer amino acid metabolites dysregulated compared to the parent grains, which exhibited intensified nitrogen absorption as a mechanism to fight PFOS stress (e.g., simultaneous increases in amino acid metabolites in arginine biosynthesis such as ornithine, citrulline, glutamine, allantoic acid, and urea). This observation reflects the removal of PFOS stress in the second-generation study.

#### Citric Acid Cycle (TCA Cycle) Metabolites

Prior exposure to PFOS (25–0 mg/kg PFOS) disturbed the synthesis of two metabolites from the citrate cycle (TCA cycle) compared to the control (0–0 mg/kg PFOS). Interestingly, none of the TCA cycle metabolites was affected in the parent grains.^5^ Fumaric acid increased in abundance by 39% whereas succinic acid decreased by 25% in daughter grains harvested from 25 to 0 mg/kg PFOS compared to 0–0 mg/kg PFOS (Figure 5). Fumaric acid and succinic acid along with aspartate and asparagine are involved in the alanine, aspartate, and glutamate metabolism pathway, and this coincides with the dysregulation in abundances of glutamic acid, aspartic acid, and asparagine. TCA is the central pathway connecting almost all of the individual metabolic pathways and the most important metabolic pathway for energy supply.^36^ The current results show that prior exposure to PFOS caused adjustment to energy consumption in wheat grains, perhaps similar to the report of Chen et al.,^32^ who found enhanced fumaric acid but decreased succinic acid metabolites in rice grains exposed to the contaminant tetrabromodiphenyl ether.

#### Other Metabolites

Ferulic acid (hydroxycinnamic acid), cadaverine (biogenic polyamine), panthotenic acid (vitamin), and inositol-4-monophosphate increased in abundance in 25–0 mg/kg PFOS compared to 0–0 mg/kg PFOS by 39, 30, 40, and 39%, respectively (Table 2 and Figure 5). Panthotenic acid’s only known metabolic function is the synthesis of CoA, which is a cofactor essential for amino acid, fatty acid, and carbohydrate metabolisms.^37,38^ Thus, the enhancement in pantothenic acid abundance could feed back toward the increase in metabolites’ abundance, particularly those of sugars and lipids.^37^ Cadaverine is synthesized by many plants via the aspartate and methionine synthesis pathway from lysine.^39^ Cadaverine has been reported to protect plants from stress and pest damage,^40,41^ although some studies also showed that it can exacerbate stress.^42^ Ferulic acid is one of the most abundant phenolic compounds in wheat grains and serves vital roles in the regulation of environmental stress.^43^ It was also reported that ferulic acid synthesis in plants is directly dependent on the availability of sucrose;^43,44^ our current data also show high abundances of both sucrose and ferulic acid. Inositol-4-monophosphate also increased in abundance by 39% in 25–0 mg/kg PFOS compared to 0–0 mg/kg PFOS. Inositol-4-monophosphate is synthesized from glucose-6-phosphate and the first isomer in the series of lipid-independent sequential phosphorylation in grains to produce higher inositol phosphates (i.e., inositol hexakisphosphate), which is an important phosphorus reservoir in cereal grains.^45−47^

The generational response of plants to emerging environmental contaminants is a huge knowledge gap in the literature. We assessed the effects of previous-generation exposure to 25 mg/kg of PFOS on the performance of daughter plants grown free of the stress the parent experienced. Biochemical assays, agronomic measurement, and metabolomics analyses were used to analyze the progeny of wheat previously exposed to PFOS. The results showed that parental exposure to 25 mg/kg PFOS had no effects on the agronomic performance (e.g., growth, yield) as well as elemental concentrations of daughter plants. However, parental exposure to PFOS altered the metabolite composition of grains in subsequent stress-free generation. Metabolomics analyses showed significant dysregulations in the sugar (sucrose), amino acid (aspartate, asparagine, glutamate, glycine, and phenylalanine), lipids (linolenic acid, palmitic), TCA cycle (fumarate and succinate), and antioxidant and stress protectant (ferulic acid, cadaverine, panthotenic acid) metabolites of the second-generation plants, although these metabolites were not altered in the parent plant. Overall, PFOS exposure in the parent generation can induce specific biochemical and metabolic changes in the grains of progeny plants, even though the changes were not evident in the parent generation.
